# Diagnostic accuracy of lung ultrasound for transient tachypnea of the newborn: a meta-analysis

**DOI:** 10.3389/fped.2026.1855601

**Published:** 2026-06-24

**Authors:** Yan Niu, Dong Han, Chen Kou

**Affiliations:** Department of Neonatology, Beijing Obstetrics and Gynecology Hospital, Capital Medical University, Beijing Maternal and Child Health Care Hospital, Beijing, China

**Keywords:** diagnosis, lung ultrasound, meta-analysis, newborn, transient tachypnea

## Abstract

**Background:**

This study aimed to evaluate the accuracy and reliability of lung ultrasound (LUS) for diagnosing transient tachypnea of the newborn (TTN) through a meta-analysis.

**Methods:**

Relevant studies were retrieved from PubMed, Web of Science, the Cochrane Library, Ovid, Scopus, and Embase databases without restrictions on language or publication date, up to December 31, 2025. Studies evaluating the diagnostic performance of LUS for TTN were included. Two researchers independently extracted data and assessed study quality using the Quality Assessment of Diagnostic Accuracy Studies-2 (QUADAS-2) tool. Statistical analyses were performed using Meta-DiSc 1.4, Stata 18.0 and RevMan 5.4 software.

**Results:**

Ten studies comprising 1,747 newborns were included in the analysis. The overall methodological quality of the included studies was moderate to high. Threshold analysis indicated no threshold effect. The pooled sensitivity, specificity, positive likelihood ratio (PLR), and negative likelihood ratio (NLR) of LUS for diagnosing TTN were 0.93 (95% confidence interval [CI]: 0.78–0.98), 0.99 (95% CI: 0.97–1.00), 81.64 (95% CI: 26.19–254.45), and 0.07 (95% CI: 0.02–0.24), respectively. The pooled diagnostic odds ratio (DOR) was 1113.94 (95% CI: 143.77–8631.13), and the area under the curve (AUC) was 0.97. Meta-regression demonstrated that LUS exhibited significant diagnostic accuracy for TTN.

**Conclusion:**

LUS is a promising modality for diagnosing TTN; however, its diagnostic accuracy varies considerably depending on study design and diagnostic criteria. Further studies are warranted to validate the utility of LUS in the diagnosis of TTN.

## 1 Introduction

Respiratory distress is one of the most common critical conditions in newborns and requires timely intervention. Transient tachypnea of the newborn (TTN), a self-limiting respiratory condition caused by delayed clearance of fetal lung fluid, is a major cause of neonatal respiratory distress. Previous studies have reported that TTN accounts for approximately 33% to 50% of cases of neonatal respiratory distress (1). Although symptoms are generally transient and typically resolve within 24–48 h, severe cases may necessitate high-concentration oxygen inhalation or even mechanical ventilation (2). In clinical practice, distinguishing TTN from other causes of neonatal respiratory distress—such as neonatal respiratory distress syndrome (NRDS), meconium aspiration syndrome (MAS), and pneumonia—is crucial, as these conditions differ in treatment strategies and clinical outcomes (3). Currently, the diagnosis of TTN primarily relies on medical history, clinical manifestations, arterial blood gas analysis, and chest x-ray (CXR). However, CXR is a relatively inconvenient imaging modality characterized by considerable inter-observer and intra-observer variability, and it exposes neonates to ionizing radiation (4). In recent years, the use of LUS has rapidly expanded in the diagnosis and differentiation of neonatal lung diseases (5, 6). Unlike imaging of other organs, LUS integrates both real anatomical structures and artifacts generated by the ultrasonic beam at air-fluid interfaces. According to existing literature, the main ultrasonic features of TTN include the double lung point (DLP), characterized by a transition in echoes between the upper and lower lung fields with a distinct demarcation point; alveolar-interstitial syndrome (AIS), defined as the presence of three or more B-lines within a lung field; compact B-lines, where dense B-lines obscure the acoustic shadows of the ribs across the entire scanned area; and pleural line abnormalities. Compared with conventional CXR, LUS offers advantages including ease of operation, real-time monitoring capability, and absence of radiation exposure (7). Although LUS cannot replace CXR, its use can reduce reliance on CXR in clinical practice. The purpose of this systematic review and meta-analysis was to evaluate the diagnostic accuracy of LUS for TTN. Although previous meta-analyses have assessed the diagnostic accuracy of LUS for TTN, several aspects remain to be further elucidated (3, 8, 9), this article included more high-quality studies and case numbers, making the research results more convincing.

## 2 Methods

This study was conducted in accordance with the Preferred Reporting Items for Systematic Reviews and Meta-Analyses (PRISMA) guidelines (10).

### 2.1 Data sources and search strategy

The authors searched six electronic databases: PubMed, Web of Science, the Cochrane Library, Ovid, Scopus, and Embase. The final search was conducted on December 31, 2025, with no restrictions on publication date, language, or country. Boolean operators (AND/OR) and controlled vocabularies were employed to reduce the number of irrelevant retrieved research results, while maximizing the search scope. Both Medical Subject Headings (MeSH) combinations and free-text terms were used, including: “Transient Tachypnea” or “TTN” or “wet lung”, and “Newborn” or “Neonatal” or “Infant”, and “Ultrasound” or “Ultrasonography” or “Ultra sound”.

### 2.2 Inclusion and exclusion criteria

The inclusion criteria were as follows: (1) studies evaluating the diagnostic accuracy of LUS for TTN (Cases with analogous clinical features were excluded); (2) studies conducted in human newborns, including term and preterm infants; (3) studies reporting sufficient data to construct 2 × 2 contingency tables. The exclusion criteria were as follows: (1) case reports, letters, editorials, comments, and reviews; (2) insufficient data to construct 2 × 2 tables; (3) duplicate publications.

### 2.3 Data extraction

Based on the predefined inclusion and exclusion criteria, two authors independently performed literature screening and data extraction. Any disagreements were resolved by consultation with a third author. Data were extracted using standardized forms. For each eligible study, the following information was collected: last name of the first author, year of publication, country, study design, sample size, blinding, mean gestational age of the newborns, reference standard for TTN diagnosis, LUS operator specialty, LUS equipment, LUS diagnostic criteria, time interval between CXR and LUS, and the numbers of true positives (TP), true negatives (TN), false positives (FP), and false negatives (FN) for LUS in diagnosing TTN.

### 2.4 Literature quality assessment

Quality assessment was independently performed by two authors using the Quality Assessment of Diagnostic Accuracy Studies-2 (QUADAS-2) tool (11). The quality of each included study was assessed by evaluating the risk of bias across four domains and clinical applicability across three domains. The four domains for risk of bias were: (1) patient selection; (2) index test; (3) reference standard; and (4) flow and timing. Clinical applicability was evaluated for the first three domains. Quality assessment was conducted using RevMan 5.4 software.

### 2.5 Statistical analysis

Spearman's correlation coefficient was performed via Meta-DiSc 1.4 to test for threshold effect. A *P*-value above 0.05 suggested no statistically significant threshold effect. Statistical analyses were performed using Stata 18.0, following standard methodological procedures for diagnostic meta-analysis. Bivariate model and hierarchical summary receiver operating characteristic (HSROC) model were applied for diagnostic meta-analysis. The bivariate model pooled sensitivity, specificity and diagnostic odds ratio (DOR), with *I*^2^ statistics adopted to assess between-study heterogeneity. The HSROC model was used to generate the SROC curve and calculate the area under the curve (AUC) for comprehensive evaluation of diagnostic efficacy (12). An AUC close to 0.5 indicates poor diagnostic performance, whereas an AUC of 1.0 reflects excellent diagnostic accuracy (13). Owing to the limited sample size and the small number of included studies, Deeks' test had low power and yielded unstable results; therefore, a funnel plot was not constructed to assess publication bias (14).

## 3 Results

### 3.1 Study selection and characteristics

**Figure 1:**
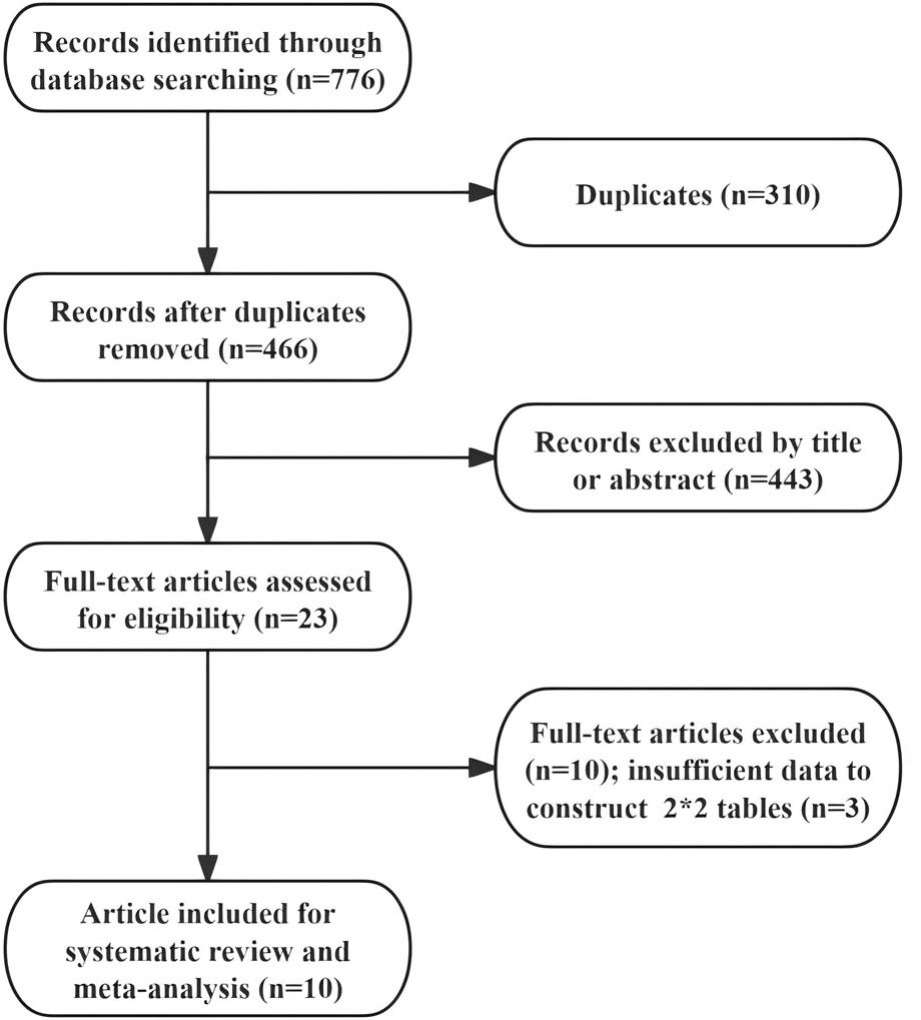
Flow diagram of the study selection process.

Based on the search strategy, a total of 776 records were identified. After removing duplicates, 466 records remained. Following screening of titles and abstracts, 443 records—including reviews, systematic reviews, and comments—were excluded. After full-text assessment, 13 records were excluded due to inconsistent research content or insufficient data to construct 2 × 2 contingency tables. Ultimately, 10 studies were included in the meta-analysis (15–24). The flow chart of the study selection process is presented in Figure 1. A total of 1,747 patients were included in this meta-analysis. The characteristics of the included studies are summarized in Table 1. Among the ten included studies, 3 (15, 16, 19) were case-control studies, and 7 (17, 18, 20–24) were cohort studies. Two studies (15, 22) used CXR diagnosis as the gold standard, and eight studies (16–21, 23, 24) combined CXR diagnosis and clinical history as the gold standard. Two of the studies (16, 18) were performed in China, three (15, 17, 22) in Italy, three (19, 23, 24) in India, one (20) in Egypt, and one (21) in France. The accuracy of included studies of LUS in diagnosing TTN is shown in Table 2.

**Table 1:** Characteristics of included studies.

Study	Origin	Study type	Sample size (n)	Gestational age^a^	Diseases diagnostic criteria	LUS diagnostic criteria	Time between CXR and LUS	LUS operator	LUS equipment	Blinding of test result interpretation
Copetti (15)	Italy	Case control study	137	34.2 ± 1.01	CXR	DLP	< 24 h	Pediatrician and cardiologist	A high-resolution 10 MHz linear probe (Megas CVX Esaote Medical Systems, Florence, Italy)and a sector 5–7.5 MHz.	Yes
Liu (16)	China	Case control study	120	27^+3^ to 36 ^+2^/28^+3^ to 36^+1^	Medical history + Clinical manifestations + Arterial blood gas analysis + CXR	DLP	< 24 h	An expert	Volusion E8 (GE Medical Systems, Milw-aukee, USA) ultrasound instruments and a linear array probe with a frequency of 9.0–12.0 MHz.	Unknow
Vergine (17)	Italy	Prospective cohort	59	33 ± 4	Clinical symptoms + CXR	Compact B-lines and DLP	< 24 h	A trained neonatologist	LUS was done with Vivid-i using a high-resolution10–12 MHz linear probe, with a dedicated preset.	Yes
Liu (18)	China	Prospective cohort	886	27^+2^ to 40^+3^/25^+4^ to 41^+3^	Clinical symptoms + CXR	DLP	< 24 h	A trained neonatologist	GE Voluson E6、E8 and Logiq C9 ultrasound equipment was used. The frequency of the linear array probe was 10–14 MHz.	Yes
Rachuri (19)	India	Case control study	94	34.5 ± 3.2/35.9 ± 2.7	Clinical symptoms + CXR	Compact B-lines and DLP	< 4 h	A research associate	Philips machine by the research associate using a linear probe of frequency 10–12 MHz and the sequence of views.	Yes
Ibrahim (20)	Egypt	Prospective cohort	65	37.3 ± 1.7/38.2 ± 1.6	Clinical symptoms + CXR	DLP	12h–24 h	A single expert	A high-resolution linear transducer with a frequency of 7–12 MHz (Philips HD7).	Yes
Grimaldi (21)	France	Prospective cohort	52	33 ± 7.3	Clinical symptoms + CXR	Lung AIS and DLP	< 3 h	Six senior neonatologists	A Philips HD100 device and one linear 5–12 MHz transducer.	Yes
Corsini (22)	Italy	Prospective cohort	134	30 ± 1/39 ± 3	CXR	Compact B-lines and DLP	< 24 h	An experienced and one from a novice sonographer.	A Philips CX50 ultrasound machine using a frequency of 10–12 MHz linear transducer.	Yes
Srinivasan (23)	India	Prospective cohort	100	33 ± 1.6	Clinical examination + Laboratory testing + CXR	DLP	< 24 h	A senior radiologist	A real-time Mindray –USG machine and 7.5MHz linear transducer.	Yes
Basha (24)	India	Prospective cohort	100	33.0 ± 1.9	Clinical signs + Symptoms + Arterial blood gas analysis + CXR	DLP	< 6 h	A trained neonatologist	Philips HD7 XE and a Sonoscape S2 portable ultrasound machine with a linear transducer (6–12 MHz).	Yes

CXR, chest x-ray; DLP, double lung point; LUS, lung ultrasound; AIS, alveolar-interstitial syndrome; MHz, mega Hertz.

a Gestational age (week) ± standard deviation.

**Table 2:** Summary of sensitivity and specificity for included studies.

Study	Sample size (*n*)	TP (*n*)	FP (*n*)	FN (*n*)	TN (*n*)	Sensitivity (%)	Specificity (%)	PPV (%)	NPV (%)
Copetti (15)	137	32	0	0	105	100	100	100	100
Liu (16)	120	46	0	14	60	76.7	100	100	81.1
Vergine (17)	59	28	1	2	28	93.3	96.6	96.6	93.3
Liu (18)	886	104	34	124	624	45.6	94.8	75.4	83.4
Rachuri (19)	94	33	0	0	61	100	100	100	100
Ibrahim (20)	65	33	0	15	17	68.8	100	100	53.1
Grimaldi (21)	52	22	0	0	30	100	100	100	100
Corsini (22)	134	30	2	2	100	93.8	98	93.8	98
Srinivasan (23)	100	47	0	3	50	94	100	100	94.3
Basha (24)	100	55	2	9	34	85.9	94.4	96.5	79.1

TP, true-positive; FP, false-positive; FN, false-negative; TN, true-negative; PPV, positive predictive value; NPV, negative predictive value.

### 3.2 Primary outcome

**Figure 2:**
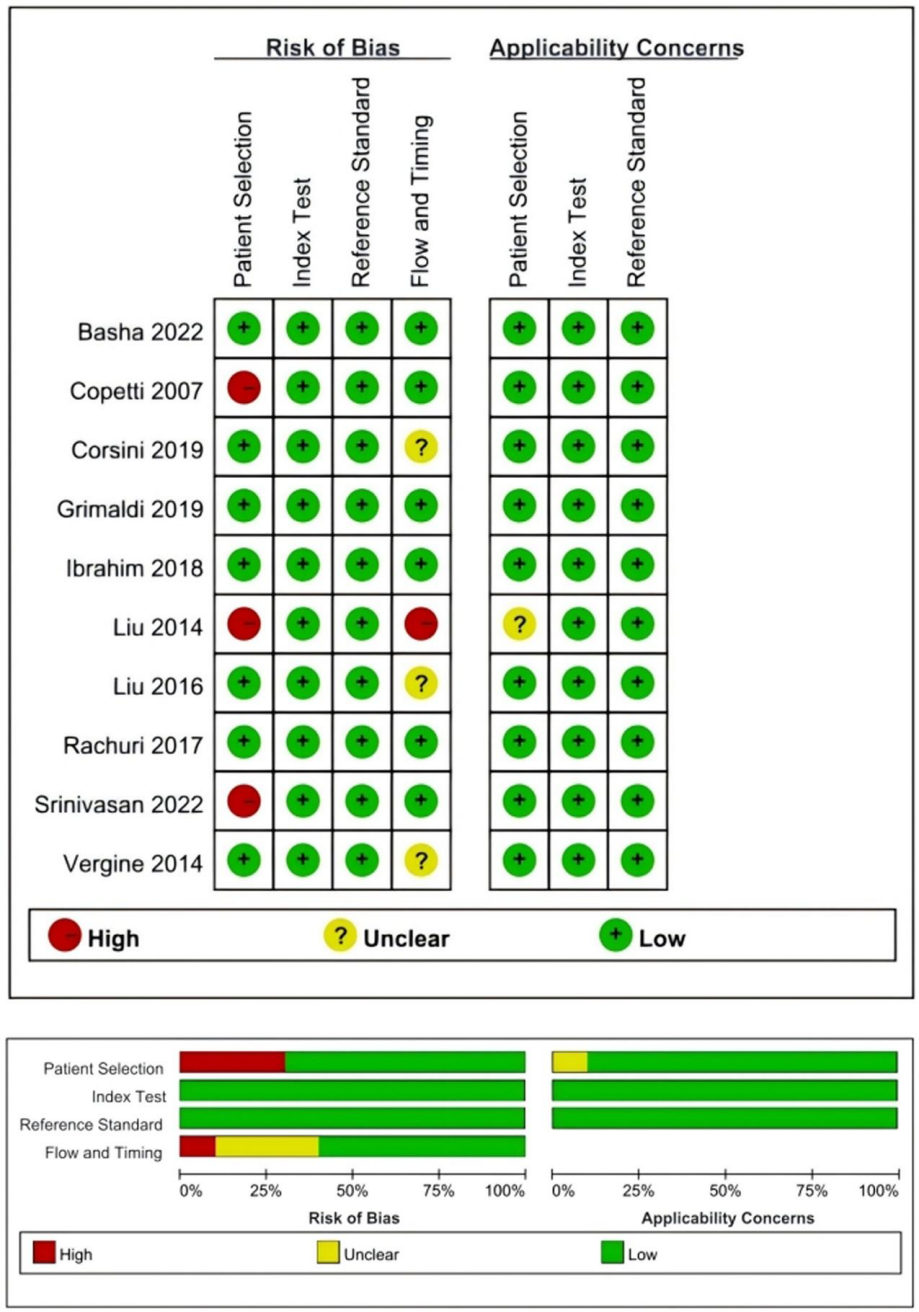
Risk-of-bias assessment of included studies.

**Figure 3:**
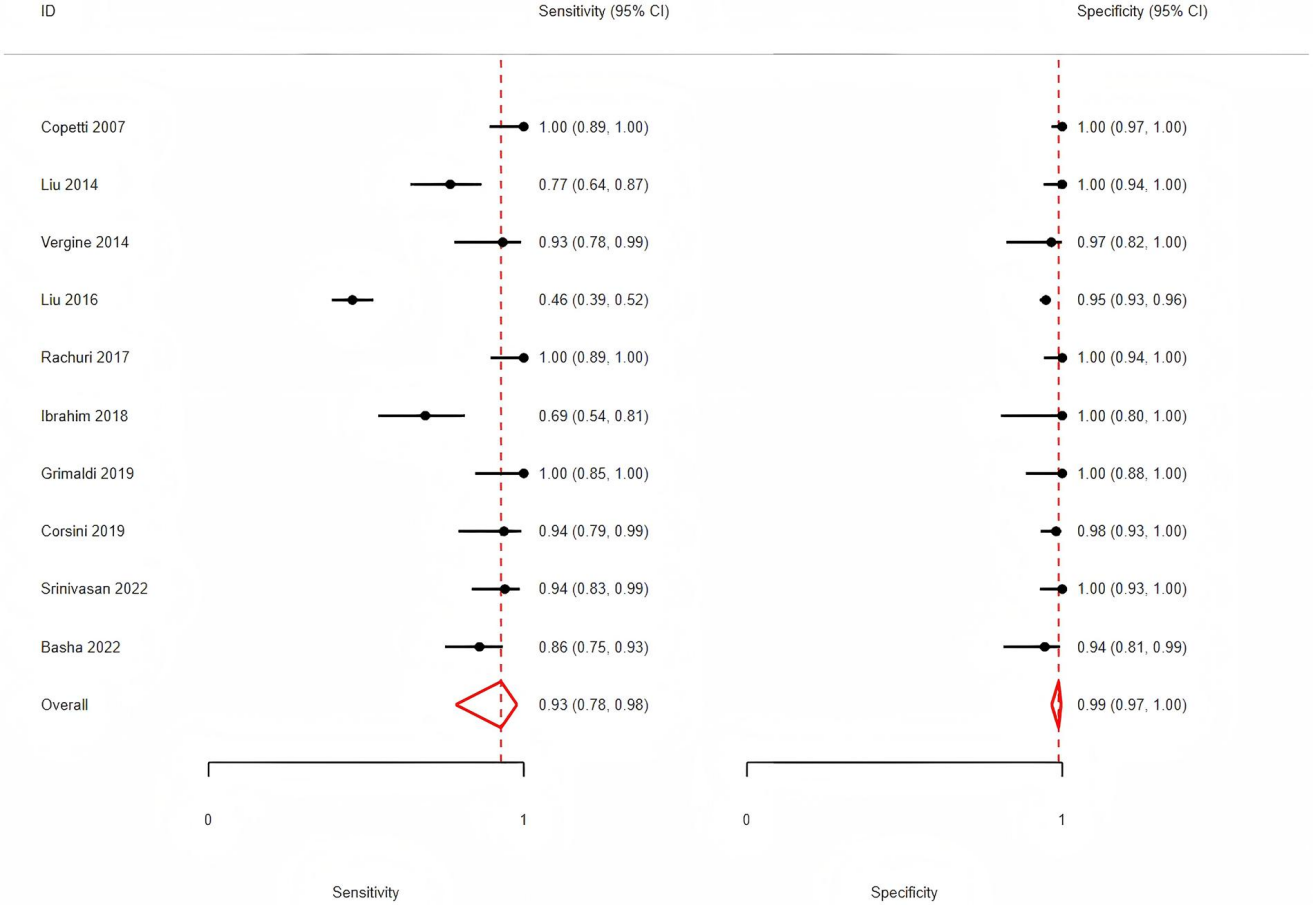
Summary analysis of sensitivity and specificity of LUS in diagnosis TTN.

**Figure 4:**
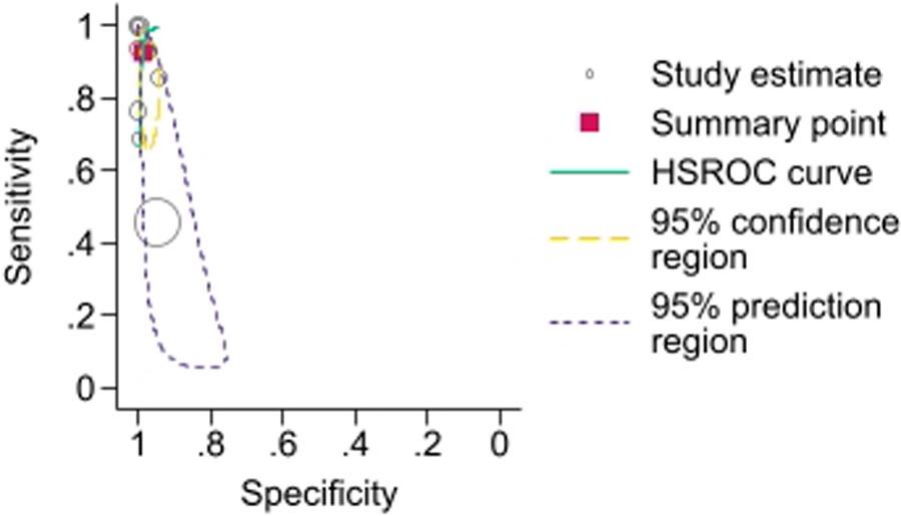
Summary pooled HSROC curve analysis of the LUS in the diagnosis of TTN.

Ten studies assessed the diagnostic accuracy of LUS for TTN in neonates. The Cohen's Kappa coefficient was calculated to be 0.85, indicating excellent inter-rater agreement. Quality assessment using the QUADAS-2 tool indicated that the overall methodological quality of the included studies was moderate to high, with detailed results presented in Figure 2. A meta-analysis of the included studies was performed, and the diagnostic performance is shown in Figures 3, 4. Threshold effect analysis revealed no statistically significant threshold effect (Spearman's correlation coefficient =–0.624, *P* = 0.054). However, heterogeneity attributable to non-threshold effects was observed among the included studies. The *I*^2^values for sensitivity and specificity, were 67.06% and 22.54%, respectively. Owing to the heterogeneity, a random-effects model was applied. The pooled sensitivity, specificity, positive likelihood ratio (PLR), and negative likelihood ratio (NLR) were 0.93 (95% CI: 0.78–0.98), 0.99 (95% CI: 0.97–1.00), 81.64 (95% CI: 26.19–254.45), and 0.07 (95% CI: 0.02–0.24), respectively. The pooled DOR was 1113.94 (95% CI: 143.77–8631.13), and the AUC was 0.97.

### 3.3 Subgroup analysis

Subgroup analysis was performed to explore potential sources of heterogeneity, with the results presented in Table 3. The findings indicated that the diagnostic criteria for LUS might be a source of heterogeneity, whereas the reference standard had a relatively small impact on diagnostic performance. Notably, using “DLP combined with B-lines” as the diagnostic criterion significantly improved the diagnostic performance of LUS.

**Table 3:** Subgroup analysis of diagnostic effect.

Subgroup	No. studies	Sensitivity			Specificity		
		value	*I*^2^ (%)	*p*	value	*I*^2^ (%)	*p*
Gold standard							
CXR	2	0.97 (0.89–1.00)	64.8	0.092	0.99 (0.97–1.00)	64.9	0.091
CXR + clinical history	8	0.69 (0.65–0.73)	94.8	< 0.001	0.96 (0.95–0.97)	65.3	0.005
LUS diagnostic criteria							
Only DLP	6	0.66 (0.61–0.70)	95.3	< 0.001	0.96 (0.95–0.97)	76.5	< 0.001
DLP and B-lines	4	0.97 (0.91–0.99)	42.4	0.157	0.99 (0.96–1.00)	11.7	0.334
Study type							
Prospective cohort	7	0.67 (0.63–0.72)	95.0	< 0.001	0.96 (0.94–0.97)	47.9	0.073
Case control study	3	0.89 (0.82–0.94)	91.1	< 0.001	1.00 (0.98–1.00)	0	1.00

## 4 Discussion

Over the past decade, LUS has emerged as a promising diagnostic tool in neonatal intensive care units (25). Previous meta-analyses have evaluated the diagnostic accuracy of LUS for TTN; however, their reported sensitivity and specificity estimates varied considerably, and some may have overestimated the diagnostic performance of LUS (3, 8, 9). By incorporating a larger number of studies and cases, this meta-analysis provides an updated evaluation of the diagnostic performance of LUS for TTN.

The studies included in this meta-analysis evaluated the diagnostic performance of LUS for TTN using two distinct diagnostic criteria. Most studies employed the DLP alone, followed by DLP combined with B-lines. DLP is attributed to differences in the severity or nature of pathology between the upper and lower lung fields, whereas B-lines arise from the alveolar gas–liquid interface. The findings of this study indicate that the sensitivity and specificity of DLP combined with B-lines for diagnosing TTN are superior to those of DLP alone. However, DLP is not a specific LUS manifestation of TTN, as it may also occur in neonatal pneumothorax, NRDS, and MAS (18, 26). Therefore, the diagnosis of TTN using DLP combined with B-lines may be more effective than using DLP alone; however, prospective multicenter studies are still needed to establish the optimal diagnostic approach. We hope that the diagnostic criteria for LUS in TTN can be standardized in the future to further enhance diagnostic reliability.

Subgroup analysis revealed that the choice of reference standard significantly influenced the diagnostic performance of LUS for TTN, with two approaches being compared: CXR alone and CXR combined with clinical history. When CXR alone was used as the reference standard, the sensitivity and specificity were 97% and 99%, respectively. Some studies suggest that LUS is more suitable for use in the NICU than CXR owing to its advantages, such as absence of radiation exposure and ease of operation. Furthermore, it has been proposed that LUS could potentially replace CXR in the diagnosis and differential diagnosis of pulmonary diseases in the NICU (27, 28). However, LUS examination has certain limitations: (1) it involves a degree of subjectivity and is operator-dependent; (2) the overall structure of the lungs cannot be visualized at one time; and (3) ultrasound findings may overlap across different diseases, necessitating the integration of other imaging modalities and laboratory tests to achieve a more accurate diagnosis. In conclusion, we believe that LUS shows promise as a supplementary diagnostic tool; however, it cannot yet replace CXR in routine clinical practice.

This study also has several limitations. First, only 10 studies were included, with a limited sample size, which may reduce the reliability and stability of the findings. Second, among the 10 included studies, three were case-control trials (15, 16, 19). Although they helped enlarge the pooled sample size, potential selection bias between cases and controls might exist, which could overestimate the sensitivity and specificity of LUS. Third, the involvement of multiple lung ultrasound operators may have contributed to the heterogeneity observed in this study. Fourth, the meta-analysis revealed substantial heterogeneity, which may reduce the reliability of the pooled estimates and limit the generalizability of the findings. Therefore, larger-scale and higher-quality studies are warranted to further evaluate the role of LUS in diagnosing TTN.

## 5 Conclusion

In conclusion, LUS is a promising modality for diagnosing TTN; however, its diagnostic accuracy varies considerably depending on study design and diagnostic criteria. Further studies are warranted to validate and compare the utility of LUS in the diagnosis of TTN.

## Data Availability

The original contributions presented in the study are included in the article/Supplementary Material, further inquiries can be directed to the corresponding author.
